# Sp1-driven up-regulation of miR-19a decreases RHOB and promotes pancreatic cancer

**DOI:** 10.18632/oncotarget.3975

**Published:** 2015-05-25

**Authors:** Yonggang Tan, Hongzhuan Yin, Heying Zhang, Jun Fang, Wei Zheng, Dan Li, Yue Li, Wei Cao, Cheng Sun, Yusi Liang, Juan Zeng, Huawei Zou, Weineng Fu, Xianghong Yang

**Affiliations:** ^1^ Department of Oncology, Shengjing Hospital, China Medical University, Shenyang, P.R. China; ^2^ Department of Pathology, Shengjing Hospital, China Medical University, Shenyang, P.R. China; ^3^ Department of General Surgery, Shengjing Hospital, China Medical University, Shenyang, P.R. China; ^4^ Laboratory of Microbiology & Oncology, Faculty of Pharmaceutical Sciences, Sojo University, Kumamoto, Japan; ^5^ Department of Medical Genetics, China Medical University, Shenyang, P.R. China

**Keywords:** pancreatic cancer, miRNA, transcription factor, target gene

## Abstract

Cancer treatment alters microRNA (miRNA) expression, revealing potential therapeutic targets (oncotarget). Here we treated pancreatic cancer (ASPC-1) cells with either recombinant human endostatin (rh-endostatin) or gemcitabine. Then high-throughput sequencing assay was performed to screen for altered miRNAs. Both treatments decreased levels of MiR-19a. We found that miR-19a stimulated cell proliferation, migration, invasion *in vitro* and tumor growth *in vivo*. High levels of miR-19a correlated with poor prognosis in patients. Ras homolog family member B (RHOB) was identified as a direct target of miR-19a. Furthermore, RHOB was down-regulated in human pancreatic cancer samples. Restoration of RHOB induced apoptosis, inhibited proliferation and migration of ASPC-1 cells. SP-1 was identified as an upstream transcription factor of miR-19a gene, promoting miR-19a transcription. Rh-endostatin decreased miR-19a expression by down-regulating SP-1. These findings suggest that miR-19a is a potential therapeutic target in pancreatic cancer.

## INTRODUCTION

Currently, there is no effective treatment for metastatic pancreatic cancer [1]. Anti-angiogenesis treatment is a promising modality [2]. Endostatin is an endogenous C-terminal fragment of collagen XVIII that inhibits endothelial proliferation and neovascularization [3]. Gemcitabine is used for pancreatic cancer. A combination of gemcitabine and endostatin has good therapeutic effect in pancreatic cancer-beard mice [4]. Therefore, we attempt to investigate biomarkers in pancreatic cancer correlated with Endostatin and Gemcitabine treatment.

MicroRNA (miRNA) is endogenous, short, non-coding RNA with approximately 22 nucleotides in length, which negatively regulates gene expression at transcriptional and post-transcriptional levels [5]. Some miRNAs are reported to be involved in pancreatic tumor development and angiogenesis [6]. As an anti-angiogenesis agent, rh-endostatin inhibits proliferation, migration, micro-vessels formation of human umbilical vein endothelial cells (HUVECs) [7–9]. Whether rh-endostatin can directly suppress pancreatic cancer cells and how miRNAs is involved remain unclear. Here we addressed these questions and revealed miRNA-19a as a potential oncotarget (therapeutic target).

## RESULTS

### MiR-19a was suppressed by rh-endostatin, gemcitabine and abraxane

**Figure 1:**
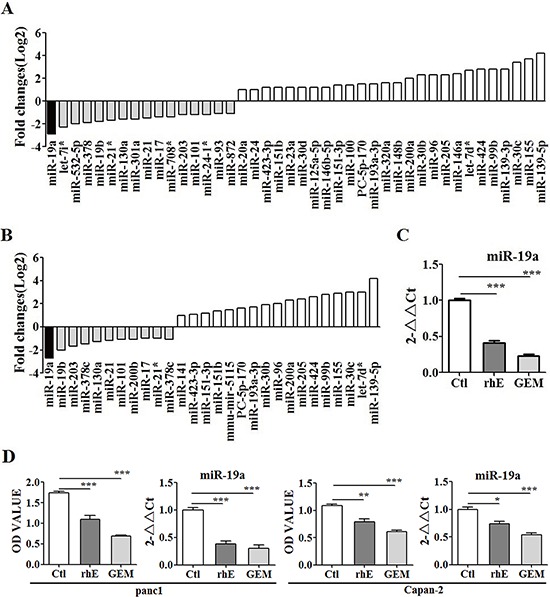
MiR-19a expression was altered significantly by both rh-endostatin and Gemcitabine **A.** High-throughput sequencing analysis showing the altered miRNA profiles after gemcitabine treatment in ASPC-1 cells. There were totally 42 miRNAs altered by gemcitabine, including 16 down-regulated miRNAs and 26 up-regulated miRNAs. MiR-19a was among the 16 down-ragulated miRNAs. **B.** The miRNA profiles were altered after Rh-Endostatin treatment in ASPC-1 cells. Totally 28 miRNAs were changed by rh-endostatin, with 11 miRNAs down-regulated and 17 miRNAs up-regulated. MiR-19a was down-regulated most significantly among the altered miRNAs. **C.** miR-19a was further identified as the same target of both gemcitabine and rh-endostatin by qRT-PCR in ASPC-1 cells. **D.** it was also confirmed that rh-endostatin and gemcitabine inhibited proliferation and simultaneously down-regulated miR-19a expression levels in Panc-1 and Capan-2 cells. *p < 0.05; **p < 0.01; ***p < 0.001.

Both gemcitabine and rh-endostatin inhibited proliferation of ASPC-1 cells (Supplementary Figure 1) and altered the miRNA profiles (Figure 1A, 1B). Totally 28 miRNAs altered by rh-endostatin and 42 miRNAs altered by gemcitabine respectively were determined by high-throughput sequencing analysis and miR-19a was down-regulated significantly in both groups, confirmed also by fluorescence quantitative RT-PCR in ASPC-1 cells (Figure 1C) and the other two pancreatic cancer cell lines (Panc-1 and Capan-2 cells, Figure 1D). The effect of abraxane on miR-19a was also explored since gemcitabine plus abraxane is a standard treatment option for pancreatic cancer patients. Abraxane inhibited cell proliferation and suppressed miR-19a expression in pancreatic cancer cell lines (Supplementary Figure 2).

### MiR-19a was over-expressed in pancreatic cancer patients

**Figure 2:**
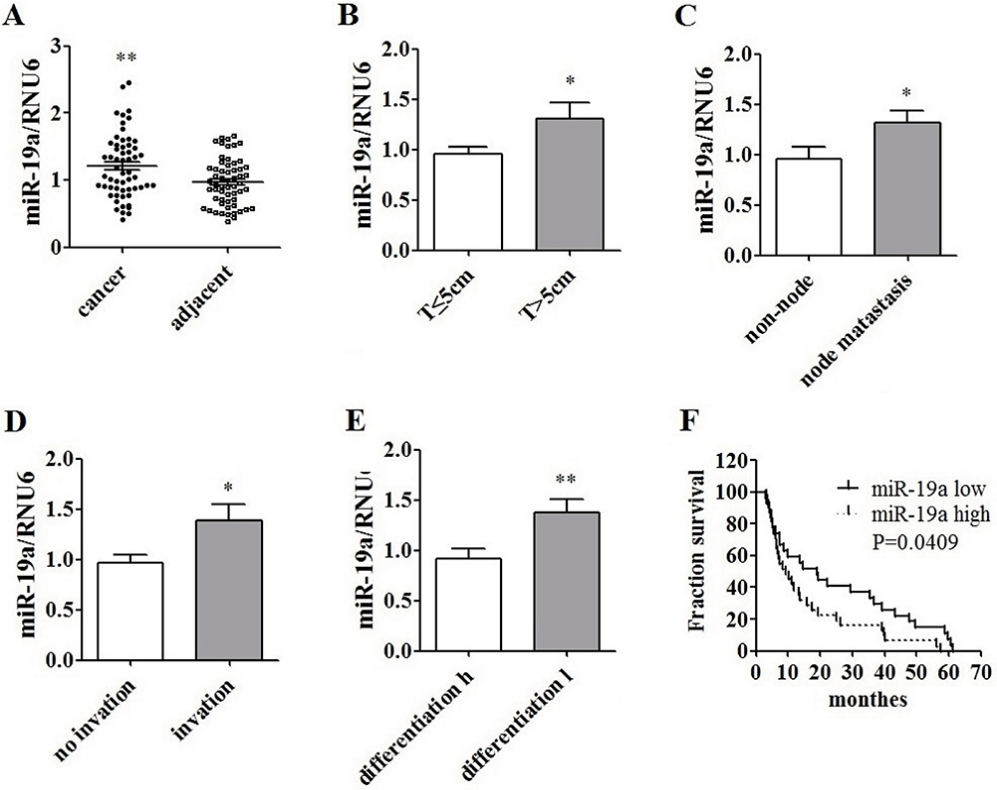
Over-expression of miR-19a in human pancreatic cancer tissues and its correlation with poor prognosis **A.** miR-19a was over-expressed in human pancreatic cancer tissues compared to that in the adjacent tissues in 58 cases. **B.** miR-19a was up-regulated in huge T phase (*T* > 5 cm) cases compared to that in cases with *T* < 5 cm. **C.** miR-19a was over-expressed in patients with lymph node metastasis compared to that in cases without lymph node metastasis. **D.** miR-19a was over-expressed in patients with local infiltration compared to the cases without local infiltration. **E.** miR-19a was over-expressed in patients with lower differentiation compared to the patients with relatively higher differentiation. **F.** High expression level of miR-19a (miR-19a levels in cancer/adjacent tissues > 1) was involved with a poor prognosis, compared to low expression level of miR-19a (miR-19a levels in cancer/adjacent tissues < 1).

Levels of miR-19a were detected in formalin fixed paraffin-embedded pancreatic cancer tissues of 58 patients (Figure 2) and 12 fresh human pancreatic cancer samples (Supplementary Figure 3A). miR-19a was over-expressed in cancer tissues compared with adjacent tissues (Figure 2A). High levels of miR-19a expression were correlated with tumor size (Figure 2B), node metastasis (Figure 2C), tumor infiltration (Figure 2D), differentiation (Figure 2E) and poor prognosis (Figure 2F).

### MiR-19a promoted progression of pancreatic cancer *in vitro* and *in vivo*

**Figure 3:**
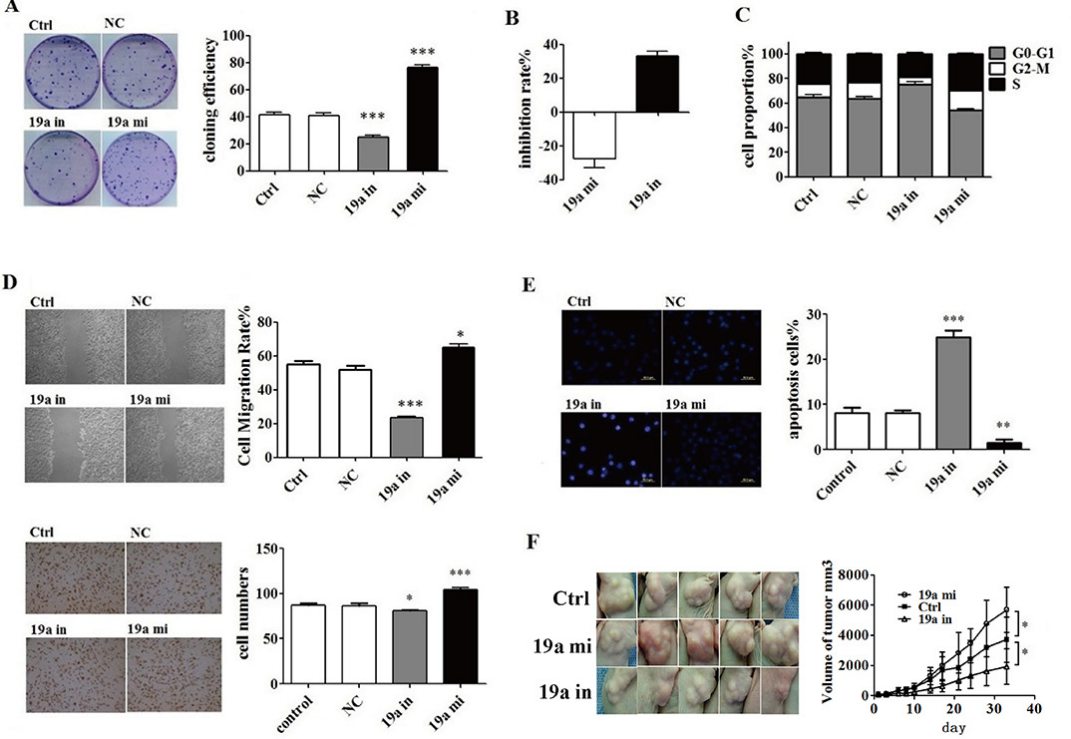
*In vivo* and *in vitro* tumor-promoting effect of miR-19a **A.** Colony formation assay indicated that miR-19a inhibitor suppressed while the miR-19a mimic promoted the colony formation of ASPC-1 cells. **B.** CCK8 test showed that miR-19a mimic promoted ASPC-1 cell proliferation, which was curbed by miR-19a inhibitor. **C.** Enhanced miR-19a increased S phase cell numbers in all cell cycle. **D.** Wound healing assays and transwell migration assays showed that miR-19a increased migration and invasion ability of ASPC-1 cell. **E.** Hochst test and Flow Cytometer (FCM) test showed that decreased miR-19a with miR-19a inhibitor induced apoptosis while increased miR-19a with miR-19a mimic inhibited apoptosis in ASPC-1 cells. **F.** MiR-19a mimics promoted pancreatic cancer growing while its inhibitors suppressed cancer growing in Female Balb/c nude mice. Values for the tumor volume (V) were determined by measuring the longitudinal cross section (L) and the transverse section (W) and then applying the formula V = (L × W^2^)/2.

miR-19a mimic and inhibitor were transfected into ASPC-1 cells separately to increase or inhibit the expression levels of miR-19a (Supplementary Figure 4A). We found that over-expressed miR-19a in ASPC-1 cells increased colony formation (Figure 3A) and proliferation (Figure 3B), increased S phase cell numbers in all cell cycles (Figure 3C), promoted cells migration and invasion (Figure 3D) and inhibited cell apoptosis (Figure 3E). The tumor-promoting effect of miR-19a *in vivo* was also confirmed (Figure 3F). The expression levels of miR-19a (Supplementary Figure 4B) and RHOB (Supplementary Figure 4C, 4D) exhibited in planted tumors illustrated that tumor progression did caused by increased miR-19a and down-regulated RHOB expression levels.

### RHOB is a direct target of miR-19a

**Figure 4:**
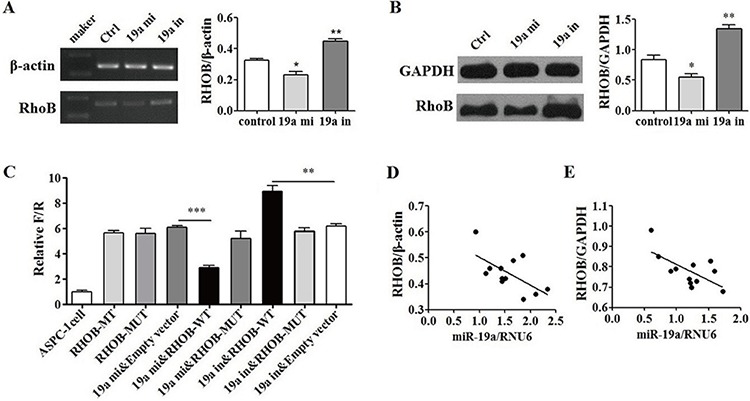
RHOB was a direct target of miR-19a **A.** RHOB mRNA and **B.** protein levels were altered by miR-19a levels inversely. Increased miR-19a expressed in ASPC-1cells with miR-19a mimic down-regulated RHOB mRNA and protein expression levels while down-regulated miR-19a with miR-19a inhibitor up-regulated the RHOB mRNA and protein expression levels in ASPC-1 cells. **C.** RHOB was determined a direct target of miR-19a by luciferase reporter assay. Increased expression of miR-19a significantly affected luciferase activity when miR-19a was co-transfected with PGL3-RHOB-WT. Conversely, the luciferase activity of a mutant reporter (PGL3-RHOB-MUT) was unaffected (*p* < 0.001). MiR-19a silencing led to a significant increase of luciferase activity in PGL3-RHOB-WT cells compared with that in the anti-NC groups (*p* < 0.01). **D.** RHOB levels were inversely related with miR-19a levels in pancreatic cancer tissues (*p* < 0.05) and **E.** in adjacent tissues (*p* < 0.05).

The functional targets prediction was performed using targetscan, pictar, miRDB and microRNA.org. RHOB was among the predicted potential targets (Supplementary Figure 5A) and miR-19a was one of the miRNAs that may target RHOB (Supplementary Figure 5B). The binding set of RHOB by miR-19a was also predicted (Supplementary Figure 5C) and the mutant set of RHOB by miR-19a was thus designed (Supplementary Figure 5D). RHOB mRNA and protein expression levels were evaluated by RT-PCR (Supplementary Table 1) and western-blot separately after transfection with mimic and inhibitor of miR-19a in ASPC-1 cells. The mRNA (Figure 4A) and protein (Figure 4B) of RHOB were both decreased in mimic group, in contrast, they were increased in inhibitor group compared with that in NC cells. Luciferase reporter assays were performed to further determine that RHOB is a direct target of miR-19a (Figure 4C). Increased expression of miR-19a significantly affected luciferase activity when miR-19a was co-transfected with PGL3-RHOB-WT. Conversely, the luciferase activity of a mutant reporter (PGL3-RHOB-MUT) was unaffected (*p* < 0.01). In addition, miR-19a silencing led to a significant increase of luciferase activity in PGL3-RHOB-WT cells compared with that in the anti-NC groups (*p* < 0.01). Furthermore, RHOB levels were inversely correlated with miR-19a levels both in pancreatic cancer tissues (Figure 4D) and in adjacent tissues (Figure 4E). These results suggest that RHOB is a direct target of miR-19a.

### RHOB inhibited cell growth, migration, and induced cell apoptosis

**Figure 5:**
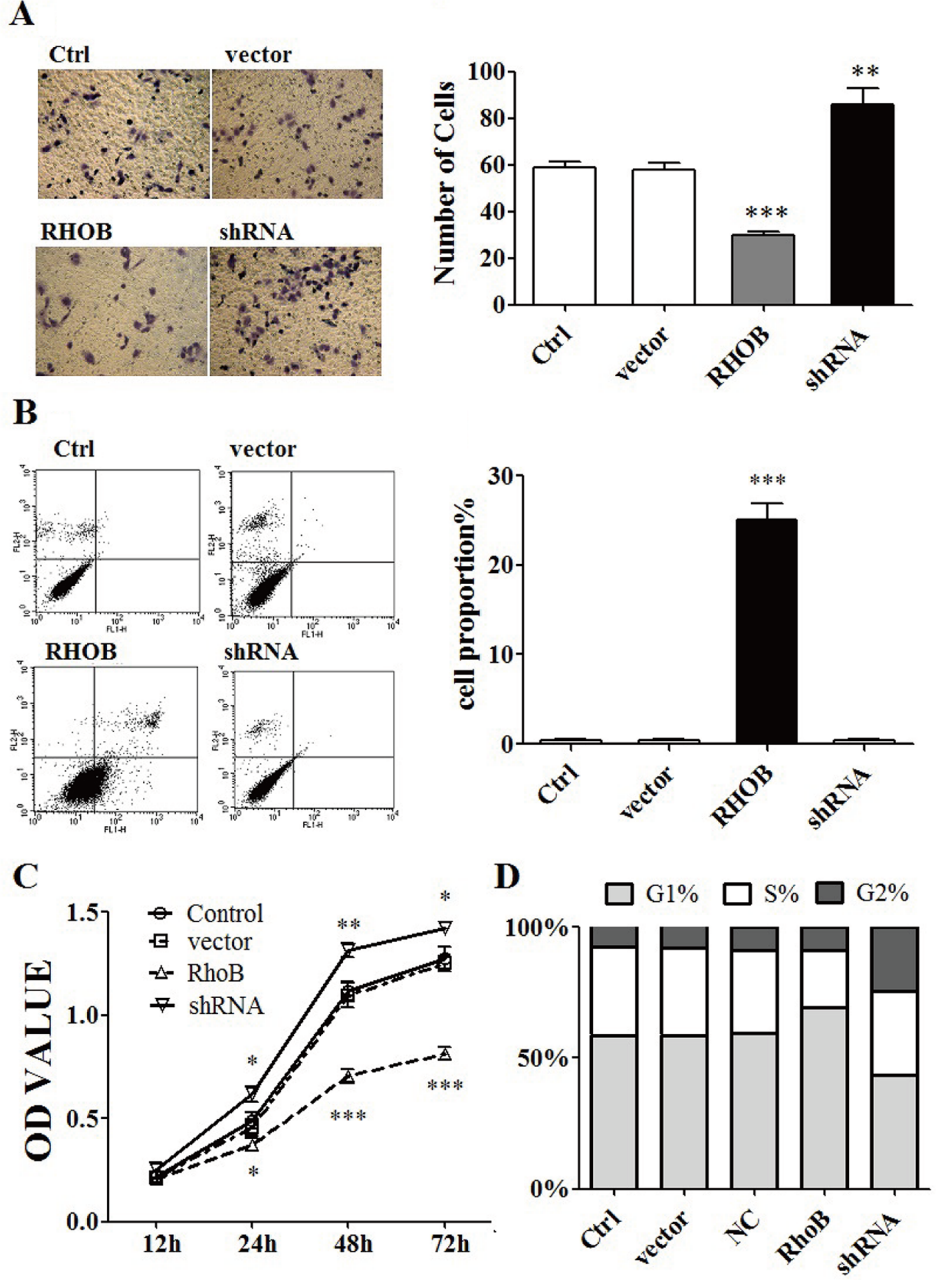
RHOB inhibited ASPC-1 cells progression **A.** Transwell migration assays showed that over-expression of RHOB decreased migration and invasion ability of ASPC-1 cells, on the contrary, down-regulation of RHOB protein level by siRNA enhanced the ability of migration and invasion in ASPC-1 cells. **B.** FCM showed that over-expression of RHOB induced ASPC-1 cells apoptosis. **C.** CCK8 test indicated that over-expression of RHOB protein level inhibited ASPC-1 cells proliferation. **D.** FCM showed that enhanced RHOB protein level decreased cell numbers of S phase in ASPC-1 cells. *p < 0.05; **p < 0.01; ***p < 0.001.

RHOB vector and siRNA plasmid (Supplementary Table 2) were constructed to increase and repress RHOB expression respectively in ASPC-1 cells (Supplementary Figure 6). Enhancement of RHOB induced cell migration, invasion (Figure 5A), apoptosis (Figure 5B), inhibited ASPC-1 cells proliferation (Figure 5C), and increased S phase cell numbers in all cell cycles (Figure 5D), down-regulation of RHOB showed the inverse effect on ASPC-1 cells. Levels of RHOB expression were detected in pancreatic cancer tissues of 12 patients (Supplementary Figure 3B).

### SP1 directly promoted miR-19a transcription

**Figure 6:**
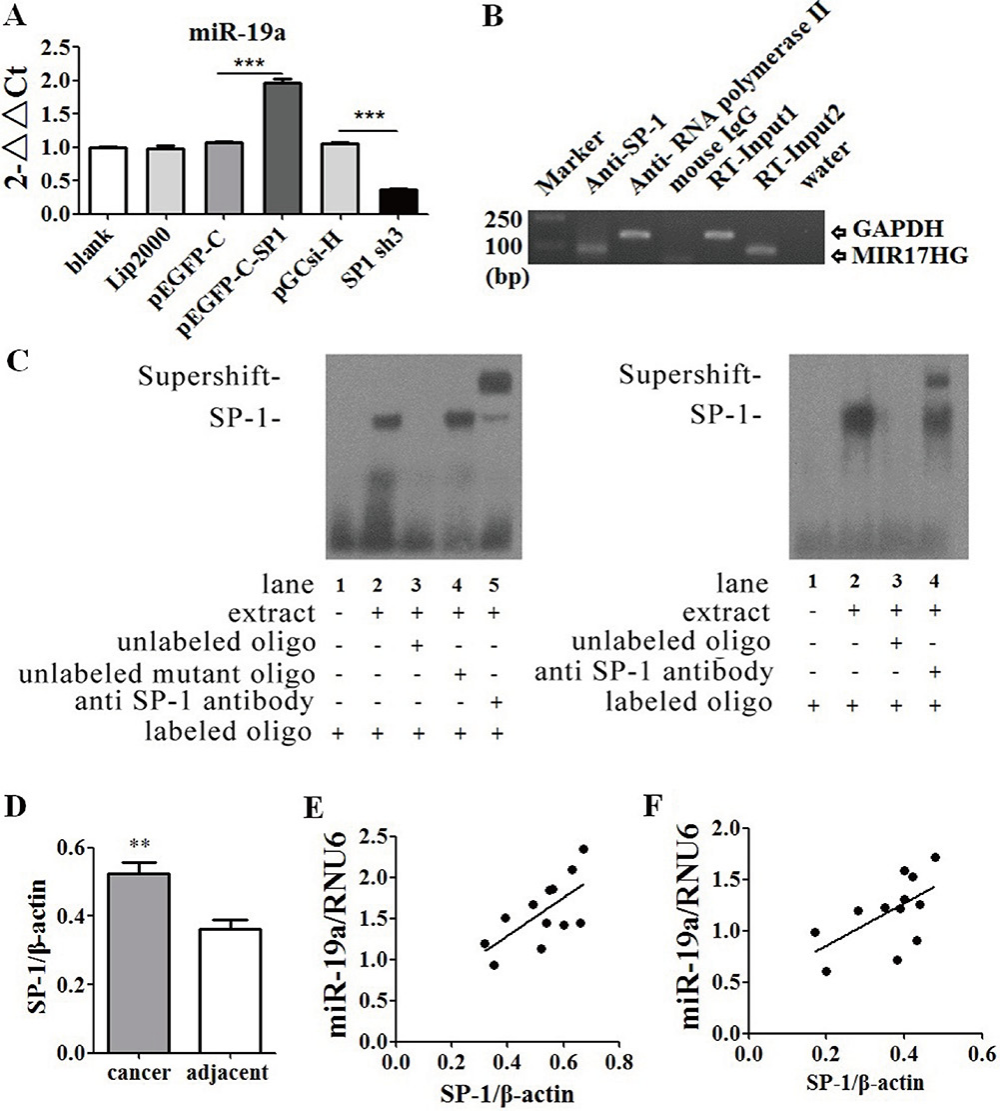
SP1 was one of the upstream transcription factors of miR-19a gene **A.** Increased SP1 expression levels up-regulated miR-19a expression levels, while decreased SP1 inhibited miR-19a expression. **B.** ChIP and **C.** EMSA identified that promotor position of miR-19a gene had binding sites for SP1. **D.** SP1 was over-expressed in human pancreatic cancer tissues than adjacent tissues at protein levels. **E.** MiR-19a expression levels were positively correlated with SP1 at protein levels in human pancreatic cancer tissues (*p* < 0.05) and **F.** in adjacent tissues (*p* < 0.05). *p < 0.05; **p < 0.01; ***p < 0.001.

To further understand the molecular mechanism for the regulation of miR-19a, the transcription factors of miR-19a were predicted using TRANSFAC^®^ 7.0 in gene regulation (http://www.gene-regulation.com/pub/databases.html) and SP1 was found to be one of the most possible transcription factors. SP1 vector and siRNA plasmid (Supplementary Table 2) were constructed to increase and repress SP1 expression respectively in ASPC-1 cells (Supplementary Figure 7). Up-regulation of SP1 in ASPC-1 cells with SP1 vector plasmid significantly increased miR-19a expression levels and down-regulated SP1 with SP1 siRNA inhibited miR-19a expression (Figure 6A). Totally 22 possible binding motifs for SP1 in miR-19a promoter region were predicted, Chromatin immune-precipitation (ChIP) assay was performed for further test but only four of them were confirmed (Figure 6B, Supplementary Table 3), which were also supported by Electrophoretic Mobility Shift Assay (EMSA) (Figure 6C). SP1 was found overexpressed in the same 12 human pancreatic cancer samples used for RHOB test (Figure 6D, Supplementary Figure 2C). MiR-19a expression was positively correlated with SP1 at protein levels in human pancreatic cancer (Figure 6E) and adjacent tissues (Figure 6F). These suggested that SP1 is one of the upstream transcription factors of miR-19a that directly promote miR-19a transcription.

### Rh-endostatin suppressed miR-19a by inhibition of SP1

**Figure 7:**
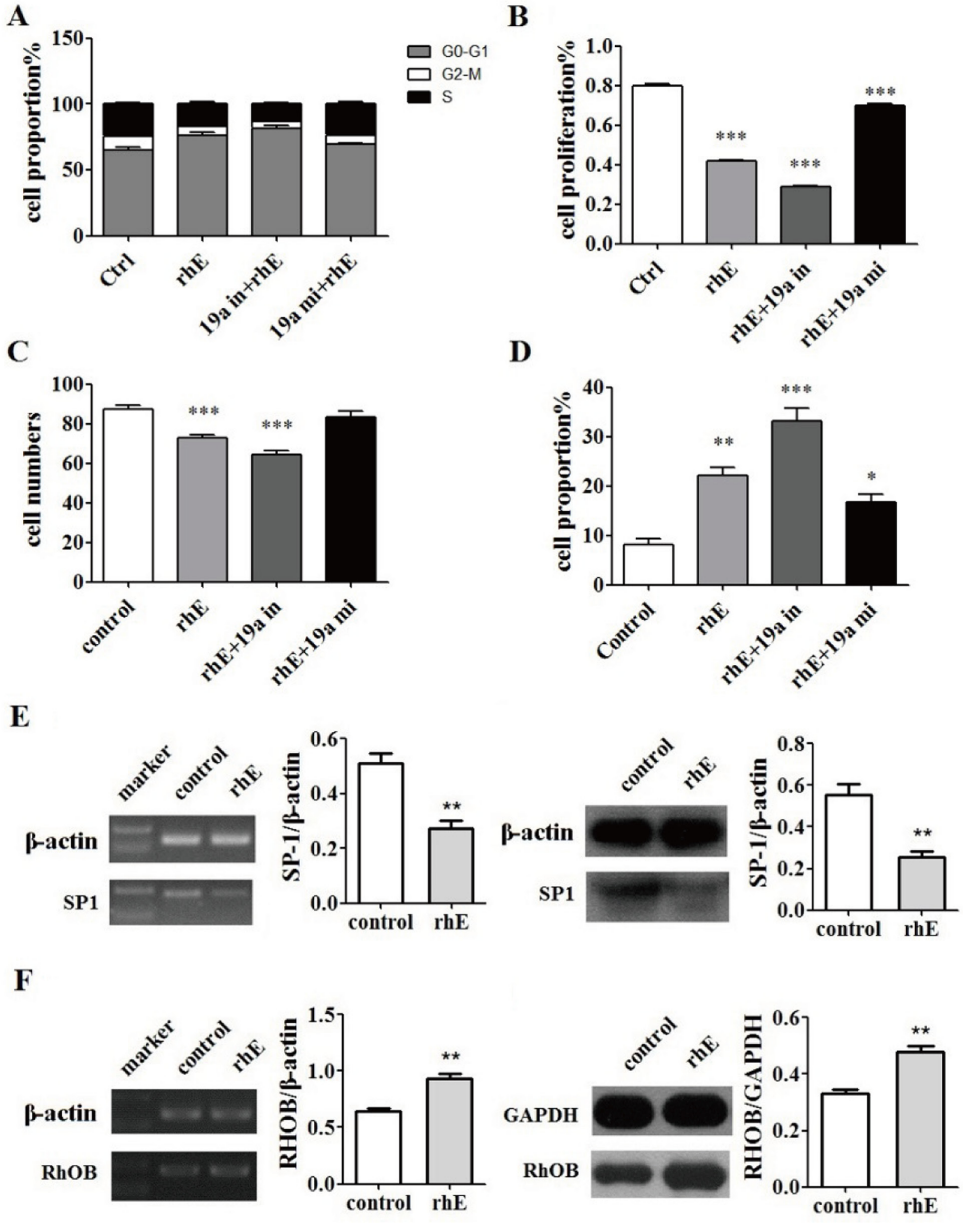
Rh-endostatin inhibited miR-19a expression by suppression of SP1 **A.** Rh-endostatin decreased cell numbers of S phase. **B.** CCK8 assays showed that rh-endostatin inhibited ASPC-1 cells proliferation and acted synergistically with miR-19a inhibitor. This effect could be partly offset by miR-19a mimic. **C.** Transwell migration assays showed that rh-endostatin reduced ASPC-1 cells migration and invasion and acted synergistically with miR-19a inhibitor, which could be completely offset by miR-19a mimic. **D.** rh-endostatin induced ASPC-1 cells apoptosis and acted synergistically with miR-19a inhibitor and inversely with miR-19a mimic. **E.** Rh-endostatin down-regulated SP1 expression at mRNA and protein levels. **F.** Rh-endostatin inhibited RHOB expression at mRNA and protein levels. *p < 0.05; **p < 0.01; ***p < 0.001.

Rh-endostatin acted synergistically with inhibitor of miR-19a in cell cycle (Figure 7A), proliferation (Figure 7B), invasion (Figure 7C) and apoptosis (Figure 7D), which were abrogated by mimic of miR-19a. SP1 was down-regulated (Figure 7E) while RHOB was up-regulated (Figure 7F) by the treatment of rh-endostatin. These findings suggest that rh-endostatin suppressed miR-19a partly by inhibition of SP1 and thus increased RHOB expression levels in pancreatic cancer cells.

## DISCUSSION

High-throughput sequencing analysis revealed 28 and 42 significantly altered miRNAs after treatment with rh-endostatin and gemcitabine. Among them, miR-19a was down-regulated more significantly than the others, including miR-21, an onco-miRNA well-known in pancreatic cancer [10–14].

The down-regulation of miR-19a by rh-endostatin and gemcitabine was identified in different pancreatic cancer cell lines. The effect of abraxane, a standard pancreatic cancer treatment, on miR-19a was explored. The IC_50_ value of abraxane on ASPC-1 cells was 4.827 μM at 72 h after treatment, consistent with previous reports [15]. Inhibition of cell proliferation and down-regulation of miR-19a by abraxane in different pancreatic cancer cell lines were confirmed, too.

As a member of miR-17–92 cluster, miR-19a is expressed in many human cancers but shows contradictory properties: namely, it can promote [16, 17] or inhibit [18, 19] cancer progression in different type of neoplasms. Recent studies have suggested that high levels of miR-19a were associated with poor prognosis [20, 21] and multidrug resistance [22, 23]. We confirmed that miR-19a was over-expressed in pancreatic cancer tissues, and was correlated with poor differentiation and prognosis, promoted cancer cell proliferation, facilitated cells migration and invasion, as well as inhibited apoptosis. These data suggested that miR-19a was an onco-miRNA and promoted progression in pancreatic cancer.

Bioinformatics search for potential target genes of miR-19a was performed by using 4 common databases, and RHOB was identified as a possible target. The assay of Dual Luciferase Reporter Gene activity confirmed that miR-19a could directly target the 3′UTR of RHOB. Our study added RHOB as one more target of miR-19a, which has been attested to target PTEN [13], SOCS1 [16], CUL5 [17], TNF-α [24] and TIMP-2 [25]. PTEN could inhibit pancreatic cancer progression through suppressing PI3K/AKT and NF-κB signal pathways [26, 27] and was targeted by different miRNAs in pancreatic cancer [28, 29]. RHOB is a tumor suppressor gene in many type of cancers [30, 31], however, RHOB, not like PTEN, is known much less in pancreatic cancer. Restoration of suppressed RHOB is a critical step for tumor regression in various types of cancers [32–37] and would be a crucial target in cancer treatment [38, 39]. Our study has demonstrated that RHOB is down-regulated in human pancreatic cancers and suppresses progression of pancreatic cancer by inhibiting proliferation, migration and invasion, as well as by inducing apoptosis. Importantly, the promoting effect of miR-19a on pancreatic cancer progression depends on inhibition of RHOB.

Sp1 is a sequence-specific DNA binding protein involved in the transcription of many important regulatory genes correlated to cancer development [40–44]. Over-expression of Sp1 usually means aggressive clinical behavior and much shortened overall survival in pancreatic cancer [45]. Sp1 is a potential therapeutic target in treatment of pancreatic cancer [46, 47]. Over-expression of Sp1 was also confirmed in pancreatic cancer tissues. Sp1 was identified as one of the upstream transcription factors that directly promote the transcription of miR-19a.

Interestingly, we also found that a high dose of rh-endostatin, an anti-angiogenesis agent, can inhibit cancer cell proliferation directly by alteration of the miRNA profiles. Rh-endostatin is now widely used for cancer treatment in Asian countries and the daily dose of rh-endostatin ranged from 3.75 to 300 mg/m^2^ is well tolerated by most of the patients and shows no significant toxicity [48–52]. The plasma concentration was about 1.35 ug/ml to 108 ug/ml for an adult with body surface area of 1.8 m^2^ and blood volume of 5 liters. Yet, the IC_50_ value of rh-endostatin on ASPC-1 cell was 1129.9 ug/ml at 48 h after treatment, an extremely high concentration. Actually, inhibition of proliferation was observed at the concentration of 250 ug/ml. Rh-endostatin inhibited miR-19a expression through down-regulating Sp1 and thus enhanced RHOB expression both in mRNA and protein levels to inhibit ASPC-1 cells proliferation, migration and invasion. Therefore it simultaneously induced cells apoptosis, and decreased S phase cells. However, it is hard for rh-endostatin to gain the effect under reported concentration in human unless it changes the dosage forms such as controlled release or liposome encapsulated formulations to meet that challenge.

In conclusion, Sp1 driven up-regulation of miR-19a promotes cancer by targeting RHOB. These findings suggest the potential of miR-19a as a therapeutic target for pancreatic cancer.

## MATERIALS AND METHODS

### Patients and pancreatic cancer tissue samples

The formalin fixed paraffin-embedded pancreatic cancer tissues specimens (tumor tissue and paired adjacent tissue) from 58 patients that underwent operation from 2004 to 2007 in Shengjing hospital, China Medical University, were collected for miRNA detection. All the 58 patients had exactly survival time. In addition, 12 fresh pancreatic cancer tissues and the corresponding normal tissues were collected immediately after resection from 2011 to 2012 in Shengjing hospital. The samples were immediately frozen at −80°C in liquid nitrogen for protein detection.

All of the patients provided written informed consent, and approval for the study was received from the Ethics Committee of China Medical University. Verification of the specimens was performed by a pathologist.

### Cell lines

Human pancreatic cancer cell line ASPC-1, Panc-1, Capan-2 were obtained from the Cell Biology Institute of Shanghai, Chinese Academy of Science and were maintained in RPMI 1640 (GIBCO, Los Angeles, CA) with 10% fetal bovine serum (Hyclone, Logan, USA), 100 units/ml penicillin and 100 μg/ml streptomycin in a humidified atmosphere at 37°C in 5% CO_2_.

### MiRNA deep sequencing

High-throughput deep sequencing was performed by a service provider LC Sciences (Houston, TX). A small RNA library was generated from the RNA sample using the Illumina Truseq Small RNA Preparation kit according to manufacturer's instructions. The purified cDNA library was used for cluster generation on Illumina's Cluster Station and then sequenced on Illumina GAIIx following vendor's instruction. Raw sequencing reads were obtained using Illumina's Sequencing Control Studio software version 2.8 (SCS v2.8) followed by real-time sequencing image analysis and base-calling by Illumina's Real-Time Analysis version 1.8.70 (RTA v1.8.70). The extracted sequencing reads was stored and then used in the standard data analysis. A proprietary pipeline script, ACGT101-miR v4.2 (LC Sciences), was used for sequencing data analysis.

### Gene transfection

Cell-based experiments were carried out by transfection of 20nM miRNA duplex (GenePharma, Shanghai, China), non-relative control RNA duplex (NC duplex, GenePharma) or small interfering RNA (siRNA, GenePharma) into the ASPC-1 cells using Lipofectamine™ 2000 in accordance with the manufacturer's procedure. The sequences of the corresponding small non-coding RNAs are as follows: miR-19a mimics: 5′-AGUUUUGCAUAGUUGCACUACA-3′; miR-19a inhibitor: 5′-UGUAGUGCAACUAUGCAAAACU-3′, mimics NC: 5′-UUCUCCGAACGUGUCACGUTT-3′, inhibitor NC: 5′-CAGUACUUUUGUGUAGUACAA-3′.

### qRT-PCR

MiRNAs were extracted from pancreatic cancer tissues, and ASPC-1 cells with miRNAs rapid extraction kit (BioTeke), and Omega E.Z.N.A miRNA kit, respectively. Detection was carried out by quantitative RT-PCR with Hairpin-it TM miRNA PCR quantitation kit (GenePharma) and specific primers for miR-19a (GenePharma) respectively, according to the manufacturer's instructions. The miR-19a expression was calculated relatively to U6 ribosomal RNA (RNU6) with U6 snRNA Real-time PCR normalization kit (GenePharma).

### Luciferase reporter assay

The 3′ UTR segment of the RHOB gene was amplified by PCR and was inserted into the vector. A mutant construct in three miR-19a binding sites of RHOB 3′UTR region was also generated using quick change site-directed mutagenesis kit (Agilent, Roseville City, CA). ASPC-1 cells were harvested for dual luciferase assay (Promega) according to manufacturer's instructions at 48 hours after co-transfection of RHOB 3′UTR or mutRHOB 3′UTR plasmid with miR-19a lentivirus vector accomplished by using Lipofectamine2000 (Invitrogen). The experiments were performed independently in triplicates.

### Chromatin immunoprecipitation

The assay for chromatin immune-precipitation (ChIP) was performed using EZ-ChIP™ Chromatin immune-precipitation kit (Millipore) according to the manufacturer's instructions. DNA pulled down by anti-SP1 antibodies was amplified by PCR. DNA from these samples was subjected to PCR analyses with primers sets for miR-19a promoter, followed by sequencing.

### Electrophoretic mobility shift assay

Nuclear proteins were extracted with a nuclear protein extraction kit in accordance with the manufacturer's instructions. The protein concentration was determined using a BCA kit. The oligonucleotide probe was 5′-TATTCGATCGGGGCGGGGCGAGC-3′ while the mutant probe was 5′-ATTGGCGGGCGGGATGGGA GGTCGGAAG-3′ and labeled with biotin. Binding reactions were performed according to the non-radioactive EMSA kit. The specificity of the DNA and protein complex was confirmed by cold competition with a 50-fold excess of unlabeled SP-1 oligonucleotides. Binding reaction, gel electrophoresis, membrane transfer and immobilization, DNA binding, chemiluminescent reaction and imaging were performed sequentially.

### Flow cytometry (FCM) analysis

Following drug treatment for various hours, the ASPC-1 cell suspension was prepared using 0.125% trypsin digestion, rinsed and centrifuged with ice-cold PBS at 1,000 rpm for 5 min. The collected cells were treated with Annexin V-FITC or PI according to the manufacturer's instruction (tube 1, unstained cells; tube 2, stained with PI; tube 3, stained with Annexin V-FITC; tube 4, stained with both Annexin V-FITC and PI) for 20 min away from the light, and Annexin fluorescence intensity was measured using FCM.

### *In vivo* experiment

Female Balb/c nude mice, 4 weeks old and weighing ~20 g were obtained from Animal Facility of China Medical University (Shenyang, P. R. China). All animals were maintained under SPF conditions and were fed sterilized water and murine chow ad libitum. All experiments were carried out according to the Guidelines of the Laboratory Protocol of Animal Handling, China Medical University.

Tumor model was established by implanting ASPC-1 cells with or without miRNA-19a mimics or inhibitors transfected (1 × 10^7^ cells) s.c. in the right axilla skin of mice. The *in vivo* study was performed on days 7–10 after tumor inoculation, when tumors were 4–5 mm in diameter and had no necrotic region. The tumor volume and body weight of the mice were measured every 2–3 days during the period of investigation. Values for the tumor volume (V) were determined by measuring the longitudinal cross section (L) and the transverse section (W) and then applying the formula V = (L × W^2^)/2.

### Statistical analysis

Statistical analysis was performed using the software Graph-Pad Prism version 5.00 for Windows, Graph-Pad Software, San Diego, California, USA, www.graphpad.com. All the data were evaluated statistically using the Student t test, the Fisher exact test, a one-way analysis of variance test, and the Pearson chi-square test. A *p* value when less than 0.05 was considered statistically significant.

## SUPPLEMENTARY MATERIALS AND METHODS FIGURES AND TABLES


