# Supplementary material for: Sp1-driven up-regulation of miR-19a decreases RHOB and promotes pancreatic cancer
Source: Oncotarget. 2015 May 25;6(19):17391–403. doi: 10.18632/oncotarget.3975 (PMC4627316; doi:10.18632/oncotarget.3975)
Supplement: Supplementary file 1 [file oncotarget-06-17391-s001.pdf]

SUPPLEMENTARY MATERIALS AND METHODS

Cell counting kit-8 test

Pancreatic cancer cells were treated with different concentration of Rh-endostatin (Simcere pharmaceutical co., LTD), Gemcitabine (Eli Lilly and Company), and Abraxane (American Pharmaceutical Partners, Inc.) separately. Cell Counting Kit-8 (CCK8) reagent from Beyotime institute of biotechnology was used for cell proliferation test, The cells were collected by centrifuging under 800 rpm for 10 min, 5×10^4 /ml cells were resuspended in the RPMI1640, seeded in 96 wells plate with 100 µl, 5000 cells per well, 5 duplicates for each well. The adherent cells were treated with endostatin at a range of concentrations of 0, 65, 125, 250, 500, 1000, 2000 (µg/ml) for 24 h, 48 h, 72 h, 96 h separately and were washed twice with PBS. Each well was then charged with 10 µl CCK-8 and the cells were incubated at 37°C for 1 h. At last absorbance values at 450 nm was measured.

miRNA extraction

miRNA were extracted with Omega E.Z.N.A miRNA kit after cells were treated by endostatin at concentration of 1100 µg/ml for 48 h, and miRNA was extracted according to manufacturer's protocol. The miRNA concentration was determinated using ultraviolet spectrophotometer.

Target & transcription factor analysis

Bioinformatics analysis for target gene was performed by targetscan (http://www.targetscan.org/index. html), pictar (http://pictar.mdc-berlin.de/cgi-bin/new_PicTar_vertebrate.cgi?species=vertebrate), miRDB (http://mirdb.org/miRDB/) and http://microRNA.org (http://www.microrna.org/microrna/home.do). Transcription factor was performed using TRANSFAC® 7.0 at gene regulation (http://www.gene-regulation.com/pub/databases. html)

miRNA transfection

The mimic and inhibitor of miR-19a were transfected into ASPC-1 cells with LipofectamineTM2000 reagent and transfection effect was confirmed by RT-PCR with TIANScript RT kit.

Reverse transcription polymerase chain reaction

Total RNA was isolated using the RNA simple Total RNA Kit (TIANGEN, Beijing, China) and complementary DNA (cDNA) was synthesized using the Super M-MLV RT Reagent Kit (BioTeke, Bejing, China). Reverse transcription polymerase chain reaction (PCR) was performed in triplicate using SYBR PremixEx Taq (TaKaRa) on an iCycler (Bio-Rad Laboratories, Hercules, CA). Gene expression levels were normalized to the internal control. The primer sequences are provided in Supplementary Table 1.

Small interfering RNA

The small interfering RNA (siRNA) target sequences used against human SP1 and RHOB were shown in Supplementary Table 2. scrambled siRNA sequence, 5′-TTCTCCGAACGTGTCACGT-3′, was used as a negative control. The oligo-nucleotides were synthesized chemically by GeneChem (Shanghai, China). SP1 and RHOB short hairpin RNA (shRNA) and control constructs pGC-pGC- SP1/RHOB -shRNA (denoted as sh-SP1 and sh-RHOB) and pGC-scramble, were generated by inserting the corresponding double-strand oligo-nucleotides into pGCsi-U6/Neo/GFP (GeneChem).

Western blot analysis

Proteins were extracted and separated via sodium dodecyl sulfate polyacrylamide gel electrophoresis (SDS-PAGE), and western blot analysis was performed according to manufacture's procedures. GAPDH was detected as internal control for normalization. The primary antibodies used in this analyses included monoclonal anti-RhoB 1:200, anti-GAPDH 1:1000, The secondary antibody was a goat anti-rabbit IgG-HRP. Proteins were then visualized using the ECL procedures (Amersham Bio-sciences, Piscataway, NJ). All experiments were performed three times.

Migration and invasion assays

Migration rate were assayed by transwell chambers and image analysis. Transfected ASPC-1 cells were

serum starved overnight and then seeded into 24-well plates with or without Matrigel-coated inserts (BD Falcon, San Jose, CA). 24 h later the cells attached to the lower surface of the insert filter were stained with crystal violet and counted. The migration rate were measured by wound healing assay according to manufacturer's instruction.

Colony formation

200 transfected cells were plated into 35 mm petri dishes and visible colonies were fixed with 4% paraformaldehyde for 20 min and stained with Wright-Giemsa dye after 14 days. Each experiment was repeated at least three times.

Hoechst

The transfected cells were plated into 12 well cell slides and cultured for 24 hrs, fixed and then stained with Hoechst staining kit.

Other chemicals frequently used in our laboratory were all products either from Sigma-Aldrich or from BD bioscience.

SUPPLEMENTARY FIGURES AND TABLES

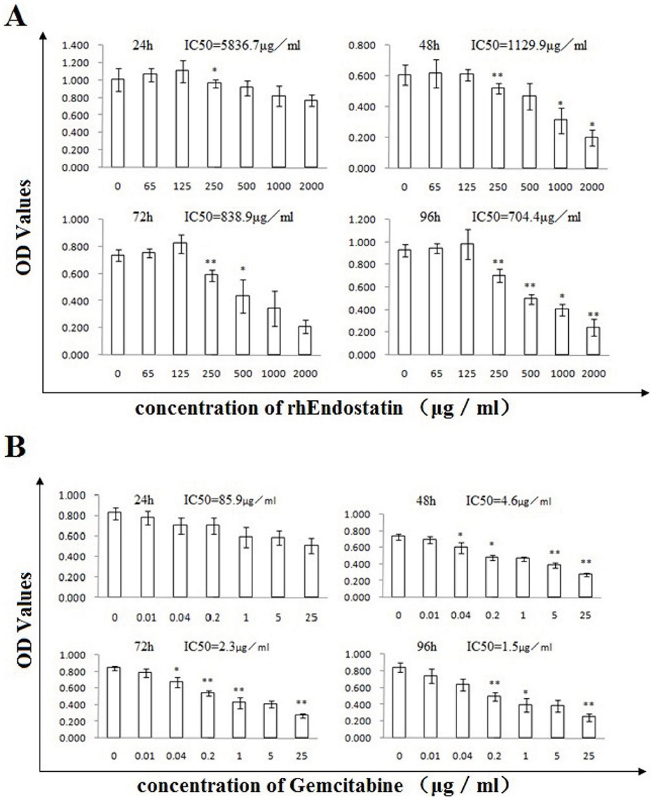

Supplementary Figure 1: CCK8 assay showed that both rh-endostatin and gemcitabine inhibited ASPC-1 cell proliferation. A. The most probable IC_{50} value of rh-endostatin on ASPC-1 cell was 1129.9 ug/ml at 48 h after treatment. **B.** The most probable IC_{50} value of gemcitabine on ASPC-1 cell was 4.6 ug/ml at 48 h after treatment. *$p < 0.05$; **$p < 0.01$; ***$p < 0.001$.

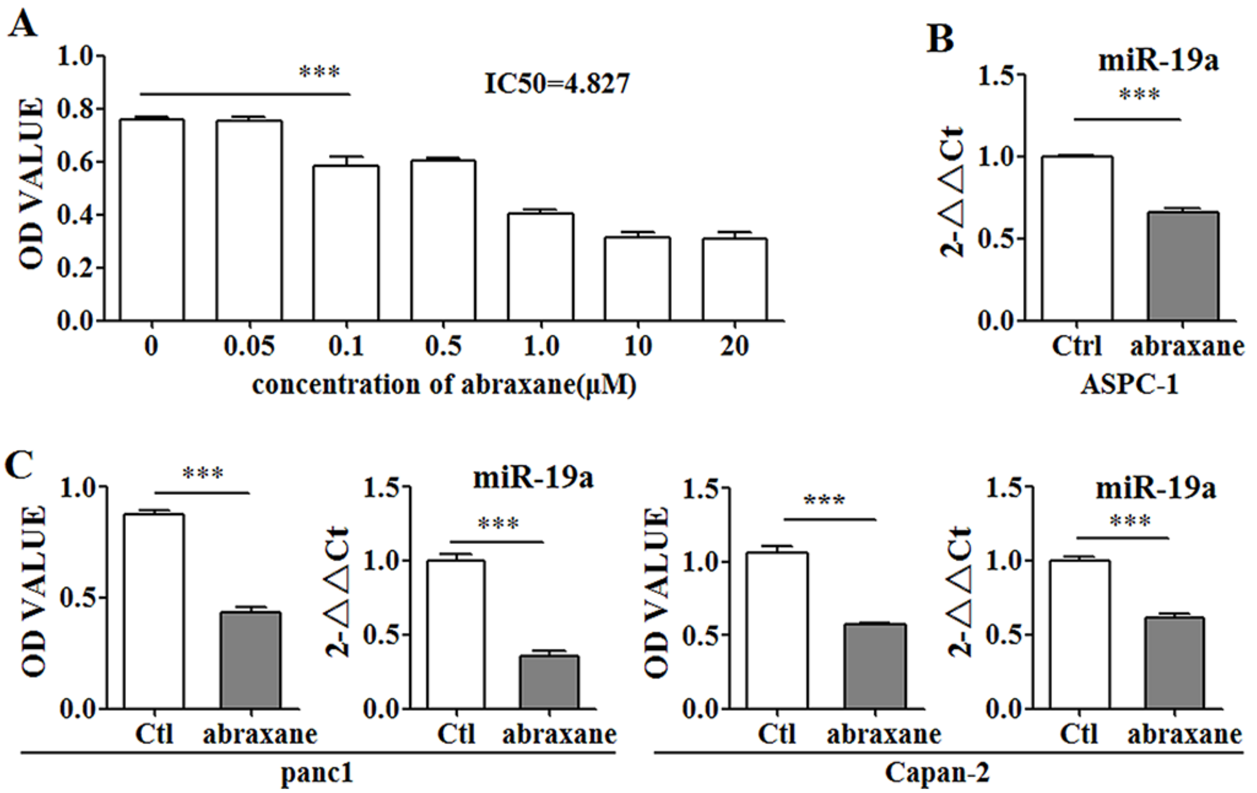

Supplementary Figure 2: Abraxane inhibited cell proliferation and suppressed miR-19a expression levels in different pancreatic cancer cell lines. A. Abraxane inhibited ASPC-1 cell proliferation and the IC_{50} value of abraxane on ASPC-1 cell was 4.827 μM at 72 h after treatment. **B.** MiR-19a was further confirmed to be down-regulated by abraxane with qRT-PCR assay in ASPC-1 cells. **C.** Abraxane inhibited proliferation and simultaneously down-regulated miR-19a expression levels in Panc-1 and Capan-2 cells.

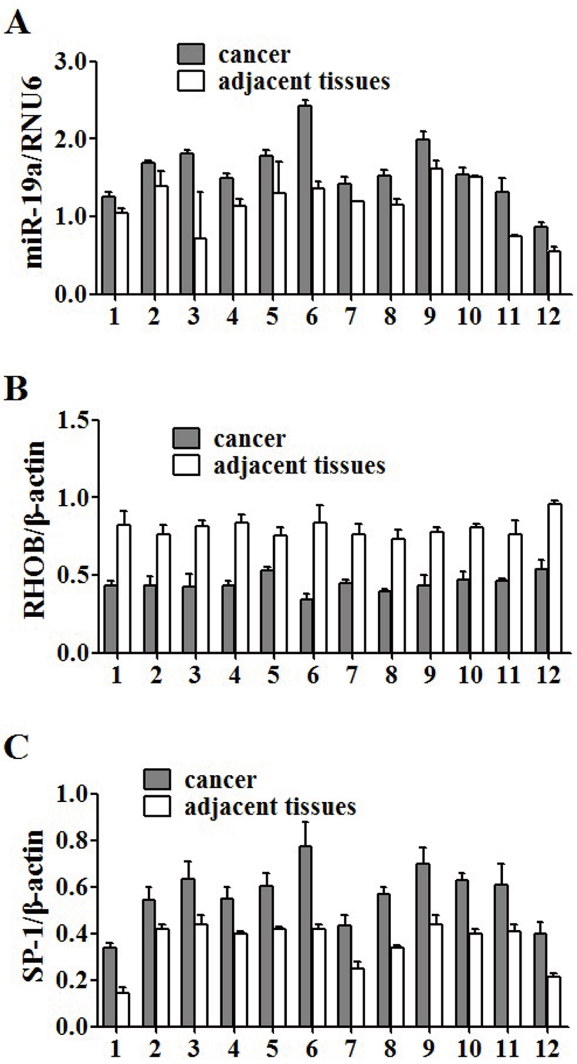

Supplementary Figure 3: The expression levels of miR-19a, RHOB and SP1 in 12 cases of fresh pancreatic cancer samples. **A.** MiR-19a was over-expressed in pancreatic cancers than adjacent non-tumor tissues. **B.** RHOB was down-regulated in pancreatic cancers than adjacent non-tumor tissues. **C.** SP1 was over-expressed in pancreatic cancers than adjacent non-tumor tissues.

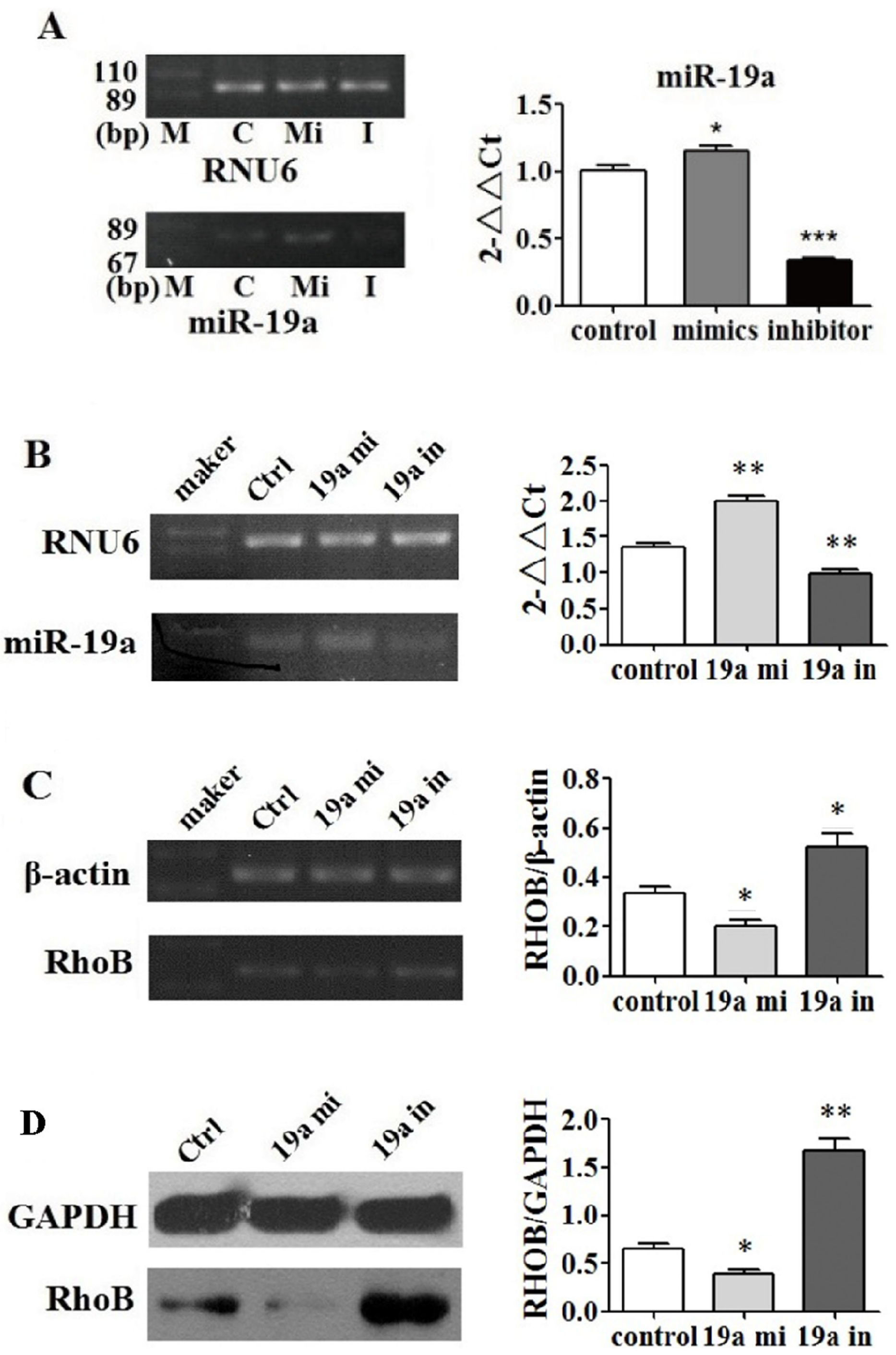

Supplementary Figure 4: MiR-19a and RHOB was altered by mimic or inhibitor of miR-19a in planted tumors. A. The effect of miR-19a mimic and inhibitor on miR-19a expression in ASPC-1 cells were identified by qRT-PCR. **B.** MiR-19a expression levels in planted tumors were detected and were proved to be still over-expressed or repressed by mimic or inhibitor of miR-19a. **C.** RHOB mRNA and **D.** protein levels in planted tumors were also determined. RHOB was down-regulated by miR-19a mimic and was increased by miR-19a inhibitor both at mRNA and protein levels.

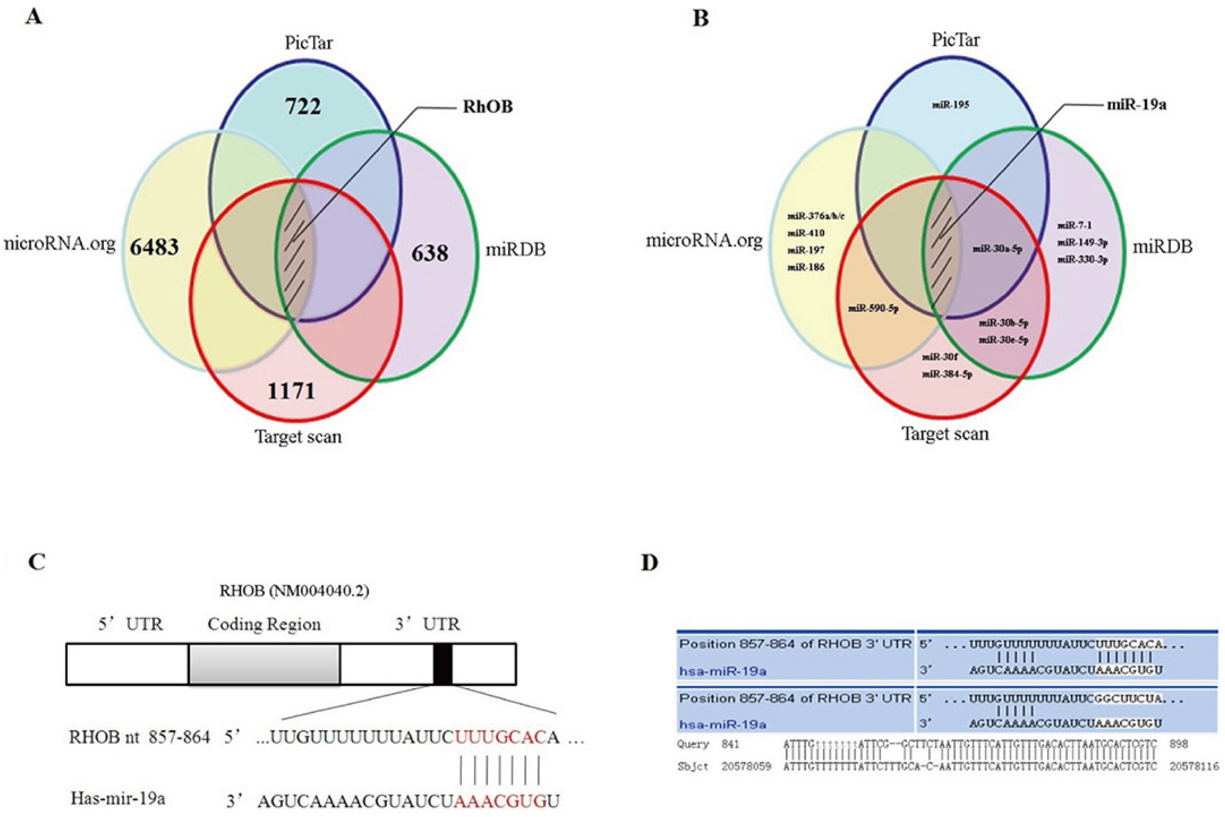

Supplementary Figure 5: Bioinformatic prediction and initial screening of potential mRNAs targeted by miR-19a. A. RHOB was predicted as one of the most probable targets of miR-19a. **B.** miR-19a was predicted as one of the miRNAs that could target RHOB by targetscan, pictar, miRDB and microRNA.org. **C.** The binding set of RHOB by miR-19a was also predicted.In complete complementation of the base of miR-19a to the 3′UTR region of RHOB mRNA was illustrated **D.** The sequence of two mutated binding site miR-137 in the 3′UTR region

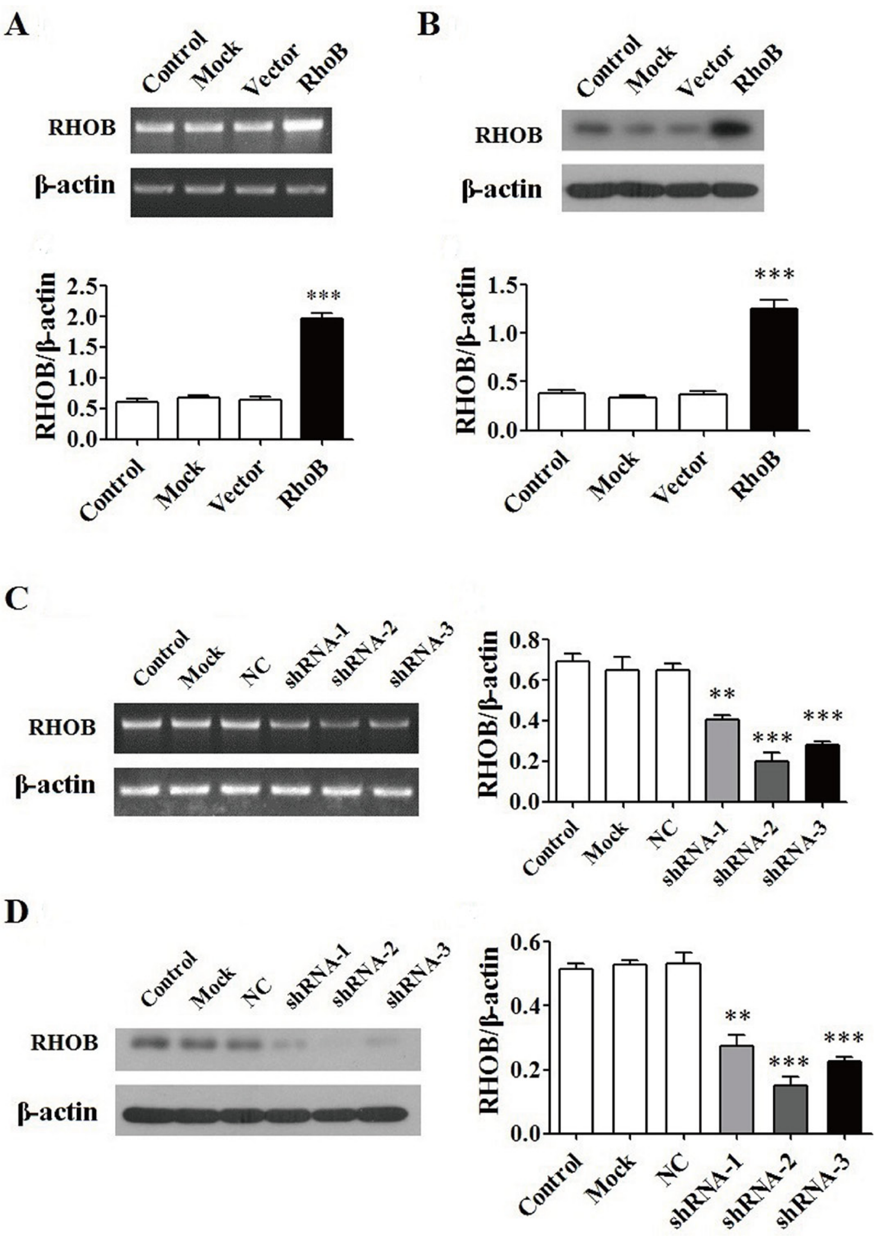

Supplementary Figure 6: RHOB over-expressed plasmid and siRNA vector construction. **A.** RT-PCR and **B.** Western-blot showed that RHOB over-expressed plasmid was successfully constructed. **C.** RT-PCR and **D.** Western-blot showed that RHOB siRNA vector was successfully constructed. Totally 3 shRNA vector was constructed and shRNA-2 had the most significant effect on the inhibition of RHOB expression which was prepared for further studies. *$p < 0.05$; **$p < 0.01$; ***$p < 0.001$.

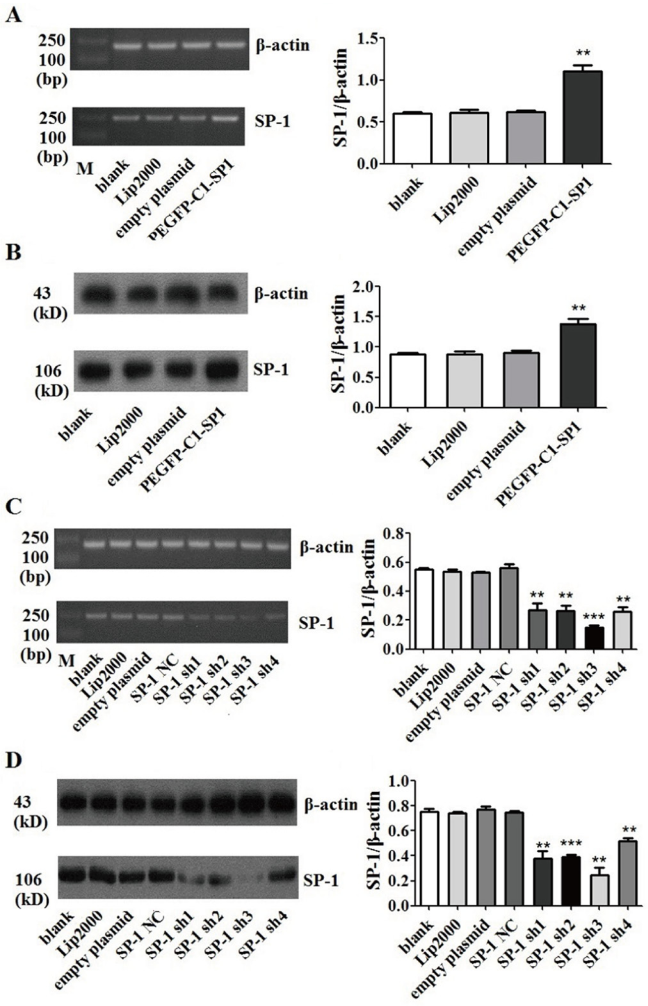

Supplementary Figure 7: SP1 over-expressed plasmid and siRNA vector construction. **A.** RT-PCR and **B.** Western-blot showed that SP1 over-expressed plasmid was successfully constructed. **C.** RT-PCR and **D.** Western-blot showed that SP1 siRNA vector was successfully constructed. Totally 4 shRNA vector was constructed and shRNA-3 had the most significant effect on the inhibition of SP1 expression which was prepared for further studies. *$p < 0.05$; **$p < 0.01$.

Supplementary Table 1: Oligonucleotide primer sets for RT-PCR

Name	Sequence (5′¬3′)	Length (bp)	Tm (°C)	Size (bp)
RHOB3’UTR-F	AAATGAGCTCGCTAGCAACTGCTGCAAGGTG	31	76.6	1286
RHOB3’UTR-R	TGTAAATGTCGACGTACCACTGGGAGGGAG	30	73.8	
RHOB-CDS-F	ATACAAGCTTCCGGCCCGCTCATGGC	26	77.1	619
RHOB-CDS-R	CGGATCCTCATAGCACCTTGCAGCAGT	27	72.5	
sp-1-ECS-F	GCGGCGAAGCTTGGATGGATGAAATGACAGC	31	80.7	2368
sp-1-ECS-R	GACTCGGTCGACTGATCTCAGAAGCCATTGCC	32	78.4	
SP-1-F	CGCTCCCAACTTACAGAACCA	21	60.3	224
SP-1-R	CAATGATGTTGCCTCCACTTCC	22	61.8	
RhoB-F	TTGAGGTGGACGGCAAGCAG	20	64.2	198
RhoB-R	TGGGCACATTGGGACAGAA	19	59.9	
β-actin-F	CTTAGTTGCGTTACACCCTTTCTTG	25	62	156
β-actin-R	CTGTCACCTTCACCGTTCCAGTTT	24	64.4	
GAPDH-F	GAAGGTCGGAGTCAACGGAT	20	58.7	224
GAPDH-R	CCTGGAAGATGGTGATGGGAT	21	60.8	

UTR, untranslated regions; CDS, Coding sequence; ECS, Enzyme cutting site; Tm, melting temperature, GAPDH, glyceraldehyde-3-phosphate dehydrogenase.
Conditions: 95°C for 5 min; 95°C for 20 s, 60°C for 20 s, and 72°C for 30 s for 30 cycles; 4°C for 5 min indefinitely.

Supplementary Table 2: The small interfering RNA (siRNA) target sequences used against human SP1 and RHOB

Name	Site	Target
RhoB-446	446	gacgtgcctgctgatcgtg
RhoB-704	704	gcacttctgtcccaatgtg
RhoB-849	849	gcctacgactacctcgagt
NC		ttctccgaacgtgtcacg
SP1-155	155	ggagttggtggcaataatg
SP1-460	460	ggaacagagtggcagcagt
SP1-568	568	ccagcaagttctgacagga
SP1-815	815	ggcctggctaataatgtac
NC		gttctccgaacgtgtcacgt

Supplementary Table 3: The binding motifs for SP1 in miR-19a promoter region

Motifs	Sequence(5′¬3′)	Primer length	Production length
SP-1-1 F	CCAAGAACGAGCCGCCGTG	19	119
SP-1-1 R	GCCTCAACGTAAATACGGACAAGC	24	
SP-1-2 F	ACATGGTCCTTCGAGGTGCC	20	136
SP-1-2 R	AGGAGAGCTTCGCGGAGGA	19	
SP-1-3 F	CTCCGGTCGTAGTAAAGCG	19	84
SP-1-3 R	GAGTAGCCGCCACCATCTTC	20	
SP-1-4 F	CGACGGAGGGAAACCTGTTGTGT	23	152
SP-1-4 R	GCCAGATGCGGCTGCCACAC	20	
